# Leisure and Problem Gaming Behaviors Among Children and Adolescents During School Closures Caused by COVID-19 in Hong Kong: Quantitative Cross-sectional Survey Study

**DOI:** 10.2196/26808

**Published:** 2021-05-07

**Authors:** Shimin Zhu, Yanqiong Zhuang, Paul Lee, Jessica Chi-Mei Li, Paul W C Wong

**Affiliations:** 1 Department of Applied Social Sciences The Hong Kong Polytechnic University Hong Kong Hong Kong; 2 Department of Health Sciences University of Leicester Leicester United Kingdom; 3 Department of Social Work and Social Administration The University of Hong Kong Pok Fu Lam Hong Kong

**Keywords:** COVID-19, leisure gaming, excessive gaming, pathological gaming, familial factors, loneliness, COVID-19 lockdown, school closure

## Abstract

**Background:**

School closures during the COVID-19 pandemic may have exacerbated students’ loneliness, addictive gaming behaviors, and poor mental health. These mental health issues confronting young people are of public concern.

**Objective:**

This study aimed to examine the associations between loneliness and gaming addiction behaviors among young people in Hong Kong and to investigate how familial factors, psychological distress, and gender differences moderate these relationships.

**Methods:**

This cross-sectional study was conducted in June 2020 when schools reopened after 6 months of school closures. Participants included 2863 children and adolescents in primary (Grades 4 to 6) and secondary (Grades 7 and 8) schools (female participants: 1502/2863, 52.5%). Chi-square tests, one-way analyses of variance, and independent-samples *t* tests were performed to compare the differences of distribution in gaming addiction behaviors across gender, age, and other sociodemographic characteristics. Multinomial logistic regression analyses were conducted to identify factors that relate to excessive or pathological gaming behaviors separately, in comparison with leisure gaming.

**Results:**

A total of 83.0% (2377/2863) of the participants played video games during the COVID-19 pandemic. The prevalence of excessive and pathological game addiction behaviors was 20.9% (597/2863) and 5.3% (153/2863), respectively. More male students had gaming addiction symptoms than female students. The multinomial logistic regressions showed that feeling lonely was associated with more problematic gaming behaviors, and the association was stronger for older female students. Low socioeconomic status, less parental support and less supervision, and poor mental health were risk factors for gaming addiction behaviors, especially among primary school students.

**Conclusions:**

Loneliness was associated with gaming addiction behaviors; the findings from this study suggested that this association was similar across gender and age groups among young people. Familial support and supervision during school closures can protect young people from developing problematic gaming behaviors. Results of this study have implications for prevention and early intervention on behalf of policy makers and game developers.

## Introduction

It is estimated that over 80% of adolescents play video games [1,2]. The prevalence of gaming disorders among adolescents is estimated to be between 1.3% and 19.9% [3], with 8.1% to 10.6% of them involved in excessive gaming and 2.3% to 2.7% involved in pathological gaming [4,5]. Young people are more likely to get involved in pathological gaming than people in other age groups [6]. Social distancing and daily routine changes due to the COVID-19 pandemic may have intensified young people’s gaming behaviors. Gaming disorders were found to be associated with poor academic performance [7,8], loneliness [9-11], and a number of health and mental health issues.

Problem gaming behaviors among physically and socially isolated young people during school closures under the pandemic have drawn the concerns of policy makers, researchers, and helping professionals. Elevated loneliness may lead to depression and suicidal ideation, and the chronic impact may last even after the pandemic. According to the self-determination theory, playing video games may provide a sense of autonomy, competence, and relatedness for most people [12]; however, uncontrolled excessive gaming may lead to gaming disorders. Notably, the linear relationship between loneliness and gaming addiction is debatable. Some studies have suggested that problem gaming behaviors were not associated with loneliness [13,14], while others provided support for their association [15-20]. Moreover, gender differences in gaming disorders, with more males having them [3,21], and inconsistent gender differences in perceived loneliness [22-24] have been observed in previous studies. Yet, few studies have examined whether there are sex differences in the association of game addiction with loneliness. As young people stay at home longer during school closures caused by COVID-19, family structure, socioeconomic status (SES) [25-28], and parenting characteristics (ie, parental support and supervision) [29,30] play a strong role in the impact of gaming behaviors on young people’s well-being and development. It is clear from the above literature that the association between gaming disorders and a sense of loneliness among young people remains uncertain.

To fill the void, this study aims to examine the prevalence of gaming behaviors during the school closure period in Hong Kong and identify factors that contribute to the association between gaming behaviors and loneliness among young people. We hypothesized that young people who were lonelier during the isolation period were more likely to be addicted to gaming and that there were age and gender differences after adjustment by familial and mental health factors.

## Methods

### Data Collection and Sample

This study adopted a cross-sectional survey design with a questionnaire that adopted a variety of validated scales from previous studies. Ethical approvals were granted by the Human Subjects Ethics Sub-Committee of the Hong Kong Polytechnic University (No. HSEARS20161222006). This study drew a stratified random sample of adolescent students from four primary schools and 13 secondary schools in Hong Kong. Students in all classes from grades 4 to 6 in primary schools and grades 7 to 8 in secondary schools were approached with an invitation letter detailing the research objectives and possible risks and benefits of participation. Parental consent was obtained and each respondent gave formal consent for his or her participation in the study by signing a consent form. We aimed to have a sample that could yield a margin of error of 0.98/√n = 1.8%. In total, 4635 students and their parents were approached and 2863 students filled in the survey, giving a response rate of 61.8%. Students completed the questionnaires in class in the presence of the research assistant and received souvenirs (worth about US $5) after the completion of the survey.

### Measures

#### Gaming Addiction

Gaming behavior was measured using the Chinese children’s version of the 7-item Game Addiction Scale (GAS) [4,31]. The GAS was conceptually developed based on the criteria for pathological gaming in the DSM-5 (Diagnostic and Statistical Manual of Mental Disorders, Fifth Edition). Some items were adapted for young adolescents (eg, “I played a game to forget my real life” was changed to “I have played games and missed planned work”) [31]. Responses were measured on a 6-point Likert scale, with scores ranging from 1 (strongly disagree) to 6 (strongly agree); scores of 4 (slightly agree) to 6 (strongly agree) were taken as endorsement of the described addiction symptom. Endorsement of zero to three items was categorized as leisure gaming, endorsement of four to six items was categorized as excessive gaming, and endorsement of seven items was categorized as pathological gaming [32]. The internal consistency of this scale, as measured by Cronbach α, is .87.

#### Gaming Time and Mode

Participants were asked to report their average hours (ie, none, half hour, 1 hour, 2 hours, 3 hours, 4 hours, 5 hours, and more than 5 hours) spent playing video games each day during school closures. Participants were also asked whether their main mode of electronic gaming was multiplayer or single player [8] and to report their most-used device for gaming: (1) smartphone, tablet, or iPad; (2) computer; or (3) other.

#### Loneliness

Loneliness was measured by a single-item question: “How often do you feel lonely?” [33]. Responses were based on frequency and ranged from 0 (not at all) to 3 (nearly every day). This is a commonly used, single self-reporting format to measure loneliness in academic research studies [34]. This short question regarding loneliness was asked directly and made it easy to administer and score [35]. Higher scores indicated greater loneliness.

#### Parental Support

Parental support was measured using the 4-item family subscale of the Multidimensional Scale of Perceived Social Support (MSPSS) [36]. The word “family” in the original MSPSS was replaced with the word “parents” to assess the social support from parents only. An example item was “My parent is willing to help me make decisions.” Students responded using a 6-point Likert scale, ranging from 1 (strongly disagree) to 6 (strongly agree). Higher scores indicated higher levels of parental support. The internal consistency of this scale, as measured by Cronbach α, is .91.

#### Parental Supervision

Parental supervision was measured by three items: (1) “My parents know what I do after school,” (2) “My parents know how I spend my pocket money,” and (3) “My parents know how I spend my free time.” These items were adapted from the Parental Monitoring Scale [37]. Students responded using a 6-point Likert scale, ranging from 1 (strongly disagree) to 6 (strongly agree). Higher scores represented higher levels of parental supervision. The internal consistency of this scale, as measured by Cronbach α, is .81.

#### Depression

Depression was measured using the Patient Health Questionnaire-9 [38,39]. It consists of nine items for assessing depression symptoms in the past 2 weeks (eg, little interest or pleasure in doing things). Response options, with respect to frequency, range from 0 (not at all) to 3 (nearly every day). Higher scores represents more depressive symptoms. The internal consistency of this scale, as measured by Cronbach α, is .85.

#### Anxiety

Anxiety was measured using the Generalized Anxiety Disorder-7 scale [40,41], which is a popular tool that screens for, and measures the severity of, generalized anxiety disorder. It consists of seven items that assess general anxiety symptoms in the past 2 weeks (eg, not being able to stop or control worrying). Response options, with respect to frequency, range from 0 (not at all) to 3 (nearly every day). Higher scores represents more anxiety symptoms. The internal consistency of this scale, as measured by Cronbach α, is .92.

#### Sociodemographic Characteristics

Information collected on the sociodemographic characteristics of the participants included gender, age, grade, family structure (ie, living with both parents, living with father or mother, or living with no parent), and SES. SES was measured by collecting information about parental education, parents having a paid job or not, the number of household appliances, the number of e-learning devices, home internet accessibility, and the family having a car or not. These questions were more tangible for young children to answer in order to collect SES information [42]. Household appliances included televisions, refrigerators, microwaves, laundry washing machines, dishwashers, air conditioners, and audio-visual equipment [43]. The number of household appliances was summed up and this indicator ranged from 1 to 7. E-learning devices included desktop computers, notebook computers, iPads or other tablets, smartphones, and e-book readers. The number of devices was summed up and this indicator ranged from 1 to 5. Home internet accessibility was measured by a single-item question: “Are internet conditions at home satisfying your needs in your daily lives and in learning?”; responses ranged from 0 to 4 [44-46]. Possible responses regarding car ownership by the student’s family were 0 (no) and 1 (yes). The four variables above reflected participants’ SES; higher values indicated higher family SES.

### Data Analysis

Data were entered into SPSS Statistics software, version 24 (IBM Corp), for analyses. First, the correlations and reliabilities for major variables were established. Second, frequencies and proportions for categorical variables or mean and standard deviation for continuous variables were used to describe the characteristics of the participants and to describe excessive or pathological gaming addiction among the participants by different characteristics. Third, chi-square tests, one-way analyses of variance, and independent-samples *t* tests were used to compare the differences of distribution in gaming addictions between genders, age groups (ie, primary or secondary school students), and other sociodemographic characteristics. Missing data were handled by multiple imputation, imputing 10 sets of complete data sets with 25 iterations per imputation. Fourth, multinomial logistic regression analyses were conducted to estimate the odds ratios (ORs) with 95% CIs of different characteristics for participants engaged in excessive or pathological gaming separately from leisure gaming; different levels of adjustment were controlled to examine the robustness of the associations. In the crude model, we examined the association between loneliness and gaming. In Model 1, we included age and gender (ie, male or female). In Model 2, we included family and parental factors of participants, including family structure (ie, living with no parent, living with father or mother, and living with both parents), father’s job status (ie, with or without a paid job), mother’s job status (ie, with or without a paid job), household appliances, e-learning devices, home internet accessibility, parental support, and parental supervision. In Model 3, we additionally adjusted for covariates of students’ mental health: depression and anxiety. We conducted subgroup analyses to examine whether the associations of gaming addiction with loneliness were different among primary school students and secondary school students.

Two subgroup analyses were conducted to examine whether the associations of gaming addiction with loneliness differed by gender and were conducted by three-step multinomial logistic regressions. The regression analyses for the age subgroups were similar to the full sample analysis. In the gender subgroup analyses in the crude model, we examined the association between loneliness and gaming. In Model 1, we adjusted for family and parental factors of participants, including age, family structure, father’s job status, mother’s job status, household appliances, e-learning devices, home internet accessibility, parental support, and parental supervision. In Model 2, we additionally adjusted for covariates of students’ mental health: depression and anxiety.

Multiple imputation was used to minimize the impact of missing data of the sociodemographic factors. These factors were father’s and mother’s educational level and father’s and mother’s job status, which had more than 25% missing data due to children indicating they did not know the answers. A total of 10 imputations were generated. The pooled standardized regression coefficients were calculated across the 10-imputation data sets [47].

## Results

### Overview

Among the 2863 students participating in this study, 181 (6.3%) were in Grade 4, 208 (7.3%) were in Grade 5, 210 (7.3%) were in Grade 6, 1209 (42.2%) were in Grade 7, and 1055 (36.8%) were in Grade 8. The participants’ ages ranged from 8 to 17 years (mean 12.6, SD 1.32). The gender ratio of the participants was close to 1:1 (male: 1346/2848, 47.3%). Most participants lived with both parents (2282/2818, 81.0%), and a small proportion of them lived with either their father (109/2818, 3.9%) or their mother (324/2818, 11.5%). Their parents’ educational levels were primary school level (father: 112/1723, 6.5%; mother: 122/1766, 6.9%), middle school level (father: 1034/1723, 60.0%; mother: 1075/1766, 60.9%), and university level or above (father: 577/1723, 33.5%; mother: 569/1766, 32.2%). Many students did not know their parents’ educational levels and/or did not answer the questions (1140/2863, 39.8% missing data regarding father’s educational level; 1097/2863, 38.3% missing data regarding mother’s educational level). Around half of their parents had a paid job (father: 1905/2037, 93.5%; mother: 1429/2088, 68.4%); many students did not know their parents’ job statuses and did not answer the questions (826/2863, 28.9% missing data regarding father’s job status; 775/2863, 27.1% missing data regarding mother’s job status). A total of 693 participants out of 2541 (27.3%) indicated that their family had a car. The mean numbers of household appliances and e-learning devices were 4.80 (SD 1.48) and 2.57 (SD 1.00), respectively. The mean response score given by participants for satisfactory home internet access was 3.04 (SD 0.93).

### The Gaming Behaviors of Children and Adolescents

A majority of students in the study (2377/2863, 83.0%) admitted that they played video games every day during the school closure period for 30 minutes (305/2863, 10.7%), 1 hour (340/2863, 11.9%), 2 hours (451/2863, 15.8%), 3 hours (516/2863, 18.0%), 4 hours (376/2863, 13.1%), 5 hours (258/2863, 9.0%), and for more than 5 hours (131/2863, 4.6%). Most of them (1927/2863, 67.3%) were involved in multiplayer games while the rest (582/2863, 20.3%) played individually. Many of them used smartphones, tablets, or iPads (1996/2863, 69.7%); computers (274/2863, 9.6%); and other devices (108/2863, 3.8%) for game playing.

The findings of the GAS are shown in Table 1. In this study, 266 participants out of 2863 (9.3%) reported that they did not play any games during the school closure period, whereas 1432 students (55.2%) reported to have played games longer than intended. The most frequently reported gaming addiction symptom was *tolerance* (1432/2595, 55.2%), which was followed by *preoccupation* (1189/2597, 45.8%), *escape* (913/2587, 35.3%), and *unsuccessful attempts to stop or reduce playing* (885/2588, 34.2%). Male participants were more likely to report gaming addiction symptoms than their female counterparts, except on the third item, “I have played games and missed planned work.”

Overall, 2518 out of 2863 (87.9%) participants reported having at least one gaming addiction symptom, with 1768 (61.8%) students playing games as a leisure activity (1 to 3 symptoms), 597 (20.9%) students reporting excessive gaming addiction behaviors (4 to 6 symptoms), and 153 (5.3%) students reporting pathological gaming behaviors (7 symptoms) (Table 2; for more detailed information about participant characteristics, see Table S1 in Multimedia Appendix 1). Male students showed a significantly higher prevalence of excessive gaming and pathological gaming behaviors than females, while more females reported no gaming behaviors (χ^2^_3_=103.1; *P*<.001). Primary school students showed a significantly higher prevalence of excessive gaming behaviors than secondary school students. Secondary school students showed a significantly higher prevalence of pathological gaming behaviors than primary school students (χ^2^_3_=8.5; *P*=.04). Those who reported excessive gaming behaviors were significantly younger than those who reported leisure and pathological gaming behaviors (*F*_3_=4.03; *P*=.007).

With respect to the influence of familial and mental health factors, students reporting pathological gaming behaviors had lower home internet access rates, less parental support and supervision, and higher loneliness, depression, and anxiety levels than students reporting leisure and excessive gaming behaviors (all had *P* values <.05).

**Table 1:** Game Addiction Scale responses and DSM-5^a^ criteria.

Statement			Gave valid response (N=2863), n (%)		Disagree^b^, n (%)		Agree^c^, n (%)		DSM-5 criterion	
**I have thought all day long about playing a game.**										Preoccupation
	All participants	2597 (90.7)		1408 (54.2)		1189 (45.8)				
	Male	1314/2583 (50.9)		620 (47.2)		694 (52.8)				
	Female	1269/2583 (49.1)		783 (61.7)		486 (38.3)				
**I have played longer than intended.**										Tolerance
	All participants	2595 (90.6)		1163 (44.8)		1432 (55.2)				
	Male	1313/2581 (50.9)		547 (41.7)		766 (58.3)				
	Female	1268/2581 (49.1)		612 (48.3)		656 (51.7)				
**I have played games and missed planned work.**										Escape
	All participants	2587 (90.4)		1674 (64.7)		913 (35.3)				
	Male	1306/2573 (50.8)		848 (64.9)		458 (35.1)				
	Female	1267/2573 (49.2)		820 (64.7)		447 (35.3)				
**Others have unsuccessfully tried to reduce my time spent on games.**										Unsuccessful attempts to stop or reduce playing
	All participants	2588 (90.4)		1703 (65.8)		885 (34.2)				
	Male	1307/2574 (50.8)		823 (63.0)		484 (37.0)				
	Female	1267/2574 (49.2)		870 (68.7)		397 (31.3)				
**I have felt upset when I was unable to play.**										Withdrawal
	All participants	2586 (90.3)		1976 (76.4)		610 (23.6)				
	Male	1304/2573 (50.7)		911 (69.9)		393 (30.1)				
	Female	1269/2573 (49.3)		1055 (83.1)		214 (16.9)				
**I have had arguments with others (eg, family and friends) over my time spent on games.**										Deceiving others
	All participants	2575 (89.9)		1913 (74.3)		662 (25.7)				
	Male	1303/2561 (50.9)		896 (68.8)		407 (31.2)				
	Female	1258/2561 (49.1)		1006 (80.0)		252 (20.0)				
**I have neglected important activities (eg, school, work, and sports) to play games.**										Loss of interest
	All participants	2583 (90.2)		1958 (75.8)		625 (24.2)				
	Male	1304/2569 (50.8)		975 (74.8)		329 (25.2)				
	Female	1265/2569 (49.2)		972 (76.8)		293 (23.2)				

^a^DSM-5: Diagnostic and Statistical Manual of Mental Disorders, Fifth Edition.

^b^Answers of *disagree* were taken to mean there were no addiction symptoms.

^c^Answers of *agree* were taken to mean there were addiction symptoms.

**Table 2:** Participant characteristics in different gaming groups.

Variable			Gaming behavior (N=2863)												*P* value
			None		Leisure			Excessive		Pathological		Overall			
**Gender^a^, n (%)**															<.001
	Male (n=1346)	85 (6.3)				833 (61.9)	332 (24.7)		96 (7.1)		1346 (100)				
	Female (n=1502)	258 (17.2)				927 (61.7)	261 (17.4)		56 (3.7)		1502 (100)				
	Total (n=2848)	343 (12.0)				1760 (61.8)	593 (20.8)		152 (5.3)		2848 (100)				
**School group^a^, n (%)**															.04
	Primary (n=599)	62 (10.4)				368 (61.4)	145 (24.2)		24 (4.0)		599 (100)				
	Secondary (n=2264)	283 (12.5)				1400 (61.8)	452 (20.0)		129 (5.7)		2264 (100)				
	Total (N=2863)	345 (12.1)				1768 (61.8)	597 (20.9)		153 (5.3)		2863 (100)				
**Live with parent^a^, n (%)**															.003
	Both parents (n=2282)	283 (12.4)				1436 (62.9)	457 (20.0)		106 (4.6)		2282 (100)				
	Father (n=109)	7 (6.4)				62 (56.9)	30 (27.5)		10 (9.2)		109 (100)				
	Mother (n=324)	38 (11.7)				180 (55.6)	77 (23.8)		29 (9.0)		324 (100)				
	Neither parent (n=103)	11 (10.7)				70 (68.0)	18 (17.5)		4 (3.9)		103 (100)				
	Total (n=2818)	339 (12.0)				1748 (62.0)	582 (20.7)		149 (5.3)		2818 (100)				
**Father’s education level^a^, n (%)**															<.001
	Primary school (n=112)	13 (11.6)				67 (59.8)	24 (21.4)		8 (7.1)		112 (100)				
	Middle school (n=1034)	106 (10.3)				652 (63.1)	221 (21.4)		55 (5.3)		1034 (100)				
	University or above (n=577)	114 (19.8)				358 (62.0)	83 (14.4)		22 (3.8)		577 (100)				
	Total (n=1723)	233 (13.5)				1077 (62.5)	328 (19.0)		85 (4.9)		1723 (100)				
**Mother’s education level^a^, n (%)**															<.001
	Primary school (n=122)	17 (13.9)				71 (58.2)	27 (22.1)		7 (5.7)		122 (100)				
	Middle school (n=1075)	124 (11.5)				658 (61.2)	229 (21.3)		64 (6.0)		1075 (100)				
	University or above (n=569)	93 (16.3)				379 (66.6)	75 (13.2)		22 (3.9)		569 (100)				
	Total (n=1766)	234 (13.3)				1108 (62.7)	331 (18.7)		93 (5.3)		1766 (100)				
**Father has a paid job^a^, n (%)**															.32
	No (n=132)	17 (12.9)				75 (56.8)	31 (23.5)		9 (6.8)		132 (100)				
	Yes (n=1905)	238 (12.5)				1207 (63.4)	379 (19.9)		81 (4.3)		1905 (100)				
	Total (n=2037)	255 (12.5)				1282 (62.9)	410 (20.1)		90 (4.4)		2037 (100)				
**Mother has a paid job^a^, n (%)**															.26
	No (n=659)	98 (14.9)				397 (60.2)	135 (20.5)		29 (4.4)		659 (100)				
	Yes (n=1429)	170 (11.9)				905 (63.3)	286 (20.0)		68 (4.8)		1429 (100)				
	Total (n=2088)	268 (12.8)				1302 (62.4)	421 (20.2)		97 (4.6)		2088 (100)				
**Family owns a car^a^, n (%)**															.41
	No (n=1848)	240 (13.0)		1128 (61.0)			391 (21.2)		89 (4.8)		1848 (100)				
	Yes (n=693)	81 (11.7)		446 (64.4)			131 (18.9)		35 (5.1)		693 (100)				
	Total (n=2541)	321 (12.6)		1574 (61.9)			522 (20.5)		124 (4.9)		2541 (100)				
Number of household appliances^b^, mean (SD)			4.90 (1.34)		4.82 (1.48)			4.71 (1.51)		4.59 (1.61)		4.80 (1.48)		.07	
Number of e-learning devices^b^, mean (SD)			2.60 (1.00)		2.64 (1.01)			2.43 (0.93)		2.33 (1.02)		2.57 (1.00)		<.001	
Home internet access satisfaction score^b^, mean (SD)			3.05 (0.99)		3.10 (0.89)			2.93 (0.97)		2.76 (1.01)		3.04 (0.93)		<.001	
Age (years)^b^, mean (SD)			12.64 (1.31)		12.63 (1.30)			12.45 (1.39)		12.78 (1.23)		12.61 (1.32)		.01	
Parental support (MSPSS^c^ family subscale score)^b^, mean (SD)			4.00 (1.20)		3.94 (1.27)			3.79 (1.20)		3.63 (1.13)		3.90 (1.24)		.002	
Parental supervision score^b^, mean (SD)			4.36 (1.23)		4.22 (1.25)			4.09 (1.24)		3.87 (1.12)		4.19 (1.24)		<.001	
Loneliness score^b^, mean (SD)			0.53 (0.92)		0.47 (0.84)			0.60 (0.88)		0.75 (1.07)		0.52 (0.87)		<.001	
Depression (PHQ-9^d^ score)^b^, mean (SD)			5.52 (5.19)		4.75 (4.84)			6.73 (5.29)		8.63 (6.51)		5.44 (5.18)		<.001	
Anxiety (GAD-7^e^ scale score)^b^, mean (SD)			4.18 (4.81)		3.38 (4.66)			4.76 (5.16)		6.15 (6.29)		3.91 (4.94)		<.001	

^a^Chi-square tests were performed to compare differences in game addiction with respect to different characteristics.

^b^One-way analyses of variance were performed to compare differences in game addiction with respect to different characteristics.

^c^MSPSS: Multidimensional Scale of Perceived Social Support.

^d^PHQ-9: Patient Health Questionnaire-9.

^e^GAD-7: Generalized Anxiety Disorder-7.

### Loneliness and Gaming Addiction

In the previous 2 weeks, 176 out of 2863 participants (6.1%) felt lonely almost every day, 206 participants (7.2%) felt lonely nearly half of the days, and 538 participants (18.8%) felt lonely several days. Among those who reported loneliness, 58.4% (536/918) were females (χ^2^_3_=21.4; *P*<.001). A significant difference was found between genders (*t*_2842_=4.40; *P*<.001) but not between age groups.

Loneliness was positively associated with both excessive and pathological gaming behaviors. After adjusting for age and gender, the adjusted ORs (aORs) of loneliness increased to 1.23 (95% CI 1.19-1.26) and 1.45 (95% CI 1.38-1.53), respectively, for excessive (Table 3) and pathological gaming behaviors (Table 4). Compared to leisure gaming, being lonely increased the likelihood of excessive and pathological gaming behaviors by 23% and 45%, respectively. The aORs did not change substantially from Model 1 to Model 2, which adjusted for parenting. However, after adjusting for depression and anxiety variables, the aORs of loneliness decreased substantially, from 1.19 (95% CI 1.15-1.23) to 0.91 (95% CI 0.87-0.95) for excessive gaming behaviors and from 1.39 (95% CI 1.33-1.47) to 0.89 (95% CI 0.82-0.96) for pathological gaming behaviors. Significant associations between loneliness and gaming disorders were also found across different age and sex groups (Tables S2-S5 in Multimedia Appendix 1). Generally, being lonely was associated with a higher risk of excessive and pathological gaming behaviors, except for primary school students where there was a higher risk of excessive gaming behaviors. The magnitude of the association was relatively higher for pathological gaming behaviors, especially for girls and primary school students.

**Table 3:** Multinomial logistic regression of excessive gaming addiction (N=2863).

Variable		Odds ratio (95% CI)^a^			
		Crude model^b^	Model 1^c^	Model 2^d^	Model 3^e^
Loneliness		1.19^***^ (1.15-1.23)	1.23^***^ (1.19-1.26)	1.15^***^ (1.11-1.19)	0.91^***^ (0.87-0.95)
Age		N/A^f^	0.91^***^ (0.89-0.92)	0.90^***^ (0.88-0.92)	0.89^***^ (0.87-0.91)
**Gender**					
	Male	N/A	1.48^***^ (1.39-1.56)	1.43^***^ (1.35-1.52)	1.67^***^ (1.57-1.78)
	Female	N/A	Reference	Reference	Reference
**Live with parent**					
	Neither parent	N/A	N/A	0.72^***^ (0.61-0.85)	0.74^**^ (0.62-0.89)
	Either father or mother	N/A	N/A	1.27^***^ (1.17-1.38)	1.24^***^ (1.14-1.35)
	Both father and mother	N/A	N/A	Reference	Reference
**Father has a paid job**					
	No	N/A	N/A	1.19^**^ (1.07-1.33)	1.19^**^ (1.05-1.34)
	Yes	N/A	N/A	Reference	Reference
**Mother has a paid job**					
	No	N/A	N/A	1.06 (1.00-1.13)	1.10^**^ (1.03-1.18)
	Yes	N/A	N/A	Reference	Reference
Household appliances		N/A	N/A	1.01 (0.99-1.04)	1.01 (0.99-1.03)
e-Learning devices		N/A	N/A	0.84^***^ (0.81-0.86)	0.86^***^ (0.83-0.89)
Home internet access		N/A	N/A	0.87^***^ (0.85-0.90)	0.87^***^ (0.84-0.90)
Parental support		N/A	N/A	0.96^**^ (0.93-0.98)	1.01 (0.98-1.04)
Parental supervision		N/A	N/A	0.99 (0.97-1.02)	0.98 (0.96-1.01)
Depression		N/A	N/A	N/A	1.09^***^ (1.08-1.11)
Anxiety		N/A	N/A	N/A	1.00 (0.99-1.01)

^a^The odds ratios and 95% CIs for excessive and pathological gaming addiction behaviors were calculated separately, with leisure gaming as the reference sample.

^b^In the crude model, we examined the association between loneliness (continual) and gaming.

^c^In Model 1, we additionally adjusted for age and gender.

^d^In Model 2, we additionally adjusted for family structure, the father’s job status, the mother’s job status, household appliances (continual), e-learning devices (continual), home internet access (continual), parental support (continual), and parental supervision (continual).

^e^In Model 3, we additionally adjusted for depression (continual) and anxiety (continual).

^f^N/A: not applicable; this variable was adjusted for in subsequent models.

^**^P<.01.

^***^P<.001.

**Table 4:** Multinomial logistic regression of pathological gaming addiction (N=2863).

Variable			Odds ratio (95% CI)^a^						
			Crude model^b^		Model 1^c^		Model 2^d^		Model 3^e^
Loneliness			1.39^***^ (1.33-1.47)		1.45^***^ (1.38-1.53)		1.35^***^ (1.28-1.43)		0.89^**^ (0.82-0.96)
Age			N/A^f^		1.07^***^ (1.04-1.11)		1.07^***^ (1.03-1.11)		1.04 (1.00-1.08)
**Gender**									
	Male	N/A		2.09^***^ (1.88-2.32)		1.90^***^ (1.71-2.12)		2.37^***^ (2.09-2.69)	
	Female	N/A		Reference		Reference		Reference	
**Live with parent**									
	Neither parent	N/A		N/A		0.60^**^ (0.43-0.82)		0.69^*^ (0.49-0.96)	
	Either father or mother	N/A		N/A		1.68^***^ (1.48-1.91)		1.85^***^ (1.61-2.13)	
	Both father and mother	N/A		N/A		Reference		Reference	
**Father has a paid job**									
	No	N/A		N/A		1.21^*^ (1.01-1.46)		1.33^**^ (1.09-1.62)	
	Yes	N/A		N/A		Reference		Reference	
**Mother has a paid job**									
	No	N/A		N/A		0.98 (0.87-1.10)		1.01 (0.89-1.14)	
	Yes	N/A		N/A		Reference		Reference	
Household appliances			N/A		N/A		1.00 (0.96-1.03)		0.97 (0.93-1.01)
e-Learning devices			N/A		N/A		0.76^***^ (0.71-0.80)		0.78^***^ (0.73-0.83)
Home internet access			N/A		N/A		0.80^***^ (0.76-0.84)		0.79^***^ (0.75-0.84)
Parental support			N/A		N/A		0.97 (0.92-1.02)		1.05 (0.99-1.11)
Parental supervision			N/A		N/A		0.94^**^ (0.89-0.98)		0.97 (0.92-1.02)
Depression			N/A		N/A		N/A		1.14^***^ (1.12-1.16)
Anxiety			N/A		N/A		N/A		1.02 (1.00-1.04)

^a^The odds ratios and 95% CIs for excessive and pathological gaming addiction behaviors were calculated separately, with leisure gaming as the reference sample.

^b^In the crude model, we examined the association between loneliness (continual) and gaming.

^c^In Model 1, we additionally adjusted for age and gender.

^d^In Model 2, we additionally adjusted for family structure, the father’s job status, the mother’s job status, household appliances (continual), e-learning devices (continual), home internet access (continual), parental support (continual), and parental supervision (continual).

^e^In Model 3, we additionally adjusted for depression (continual) and anxiety (continual).

^f^N/A: not applicable; this variable was adjusted for in subsequent models.

^*^P<.05.

^*^^*^P<.01.

^***^P<.001.

### Familial Factors and Gaming Addiction

Family structure and socioeconomic factors were found to be significantly associated with gaming behaviors. Students from single-parent families (aOR 1.85, 95% CI 1.61-2.13; *P*<.001) and with unemployed fathers (aOR 1.33, 95% CI 1.09-1.62; *P*=.006) were at a higher risk of developing pathological gaming behaviors (Table 4). Higher SES reflected by more e-learning devices (aOR 0.78, 95% CI 0.73-0.83; *P*<.001) and satisfactory internet access (aOR 0.79, 95% CI 0.75-0.84; *P*<.001) were associated with fewer gaming addiction symptoms. The magnitude of the associations was higher among primary school students compared to secondary school students (Tables S2 and S3 in Multimedia Appendix 1).

Parental support was a significant protective factor for excessive (aOR 0.87, 95% CI 0.82-0.92; *P*<.001) and pathological (aOR 0.82, 95% CI 0.73-0.93; *P*=.002) gaming behaviors only among primary school students (Table S2 in Multimedia Appendix 1) but not among secondary school students. Higher perceived parental support, however, was associated with more excessive and pathological gaming behaviors among secondary school students after adjusting for depression and anxiety (*P*<.001) (Table S3 in Multimedia Appendix 1).

Perceived parental supervision was not significantly associated with gaming disorders among the whole sample, except for pathological gaming behaviors in Model 2 (aOR 0.94, 95% CI 0.89-0.98; *P*=.007) (Table 4). However, in the stratified analysis, the significant protective effect of higher parental supervision for pathological gaming behaviors was found among secondary school students (aOR 0.91, 95% CI 0.86-0.96; *P*=.001), especially for female students (aOR 0.81, 95% CI 0.75-0.89; *P*<.001) (Tables S3 and S5 in Multimedia Appendix 1), but not among primary school students. In terms of gender differences, parental supervision was a significant protective factor for excessive gaming behaviors among male students only (Table S4 in Multimedia Appendix 1). On the contrary, parental supervision was a significant risk factor for excessive gaming behaviors among female students only (Table S5 in Multimedia Appendix 1).

## Discussion

### Principal Findings

It is alarming that excessive and potential pathological gaming behaviors were prevalent among adolescent students in Hong Kong when schools were closed owing to the COVID-19 pandemic. Female students in this study were more likely to report loneliness than male students, while those with excessive and pathological gaming behaviors reported more loneliness. The results showed that loneliness was positively associated with gaming addiction when adjusted for sociodemographic characteristics, but the association became negative when depression and anxiety were present. It is noteworthy that familial factors played protective roles in the development of excessive and pathological gaming behaviors only for the younger students.

A recent systematic review on addictive gaming behaviors suggests that the prevalence rates for excessive gaming range from 1.2% to 8.1% and those for pathological gaming range from 1.4% to 2.7% [3]. Empirical studies on gaming behaviors among young people in Hong Kong have been scant. One study conducted in 2014 concluded that 93.2% of 503 high school students had played video or internet games, and 15.7% had a gaming addiction [48]. Our findings indicate that the rates of excessive and pathological gaming behaviors among students are much higher than the local and international rates, and they seem to suggest that there are positive relationships between school closures during the COVID-19 pandemic and addictive gaming behaviors among adolescents. The COVID-19 pandemic definitely disrupted students’ daily routines, limited their outdoor activities, and, hence, easily enabled students to play games for leisure to reduce loneliness and to connect with others [49]. This study has affirmed the positive relationship between loneliness and gaming behaviors that was concluded by previous studies [16,17] and highlighted the situation during a pandemic when many schools are closed down accordingly. However, the conclusion that excessive gaming behaviors increased among students in Hong Kong was not substantiated as we did not have data collected from before the outbreak of COVID-19 for comparison. Nevertheless, when comparing with the findings from previous studies based on school students in Hong Kong, the prevalence of excessive gaming behaviors among the sample of this study is prominent.

It is interesting to find that although male students in this study exhibited significantly higher game addiction behaviors than their female counterparts [7,50], female secondary students were more likely to have a higher risk of pathological gaming behaviors. It is speculated that older female students are more likely to have a desire for closeness with others and dependency on peer relationships, worrying more about abandonment, loneliness, and loss of relationships [51,52] during the school closure period. They were, therefore, more likely to use online gaming as a coping strategy for maintaining social connection in dealing with adverse emotional experiences [53-57] during the isolation period. Truly, any development of prevention and intervention for addictive gaming behaviors must take the relationship between gaming and gender into account [56].

Regarding the mental health of students with gaming behaviors, students with excessive and pathological gaming behaviors were more likely to be depressed, anxious, and lonely [17,58]. However, when these variables were analyzed within multilevel models, loneliness became negatively associated with gaming addiction behaviors. We suspect that feeling depressed may decrease the motivation for socializing and decrease the motivation for gaming among both male and female students [59,60].

Familial factors play a crucial role in the association between loneliness and gaming addiction behaviors, especially for primary school students. It was found that students from single-parent families, with unemployed fathers, or with lower SES were more likely to exhibit gaming addiction behaviors. Considering how SES relates to gaming behaviors, we suspect that those with lower SES had fewer options for leisure activities (eg, music, cooking, and traveling). For primary school students but not secondary students, if their mothers had no paid job and stayed at home, they may have had more parental support and supervision that led to a lesser chance for developing gaming addiction behaviors. Younger children who are supervised and supported by their mothers have been found to be less vulnerable to loneliness and problem gaming behaviors [29,30]. In other words, age also plays an important role in the development of gaming addiction behaviors.

### Strengths and Limitations

To our knowledge, this is the first study that examines the association between loneliness and gaming addiction behaviors among young people in Hong Kong during the pandemic. Stratified analysis of age and gender subgroups provided in-depth and specific information that may aid in prevention and treatment of gaming addiction. In addition, incorporating familial and mental health factors as extensive and significant confounders in adjusted multivariable analysis may make the associations of gaming addiction and loneliness valid and robust. Also, the classroom surveys with rigorous, systematic, and consistent instructions by trained research assistants could yield more reliable data than online surveys.

Some limitations associated with this study should be noted when interpreting the findings reported above. First, the cross-sectional data generated from this study may make causal inferences difficult. Loneliness may be the cause or consequence of gaming addiction behaviors. The negative association between loneliness and gaming addiction behaviors adjusted for depression and anxiety may also be explained reversely. For instance, depressed students who play games may have reduced their feelings of loneliness. Besides, cross-sectional data make it uncertain whether gaming addiction behaviors increased during the COVID-19 pandemic. A longitudinal or panel study collecting information on gaming involvement and levels of loneliness of school students over time will assist in ascertaining the impact of various factors. Also, collecting qualitative data from target groups and their parents and teachers who are directly in touch with them can enrich the interpretations of the results. In addition, future studies may also include pre-COVID-19 gaming experience, types and frequencies of nongaming leisure activities, and levels of accessibility to outdoor facilities in the survey because they are important confounding factors that may provide contextual information of one’s gaming behaviors. Second, the self-reported responses were less able to capture multifaceted information about loneliness. However, the single-item measure of loneliness, which is well adapted to measure loneliness, has been shown to be valid in previous studies [34]. Third, the generalizability of this study’s findings is limited to upper primary school and lower secondary school students as well as context (ie, based on one city, Hong Kong). A diversified sample that includes in-school and out-of-school adolescents across cities may address this inadequacy.

### Implications and Conclusions

Despite these limitations, these findings expand our knowledge regarding gaming behaviors and loneliness among primary and secondary school students in Hong Kong during the pandemic. Findings of this study have potential public health and clinical implications. The results suggest that problematic gaming behaviors are common among young people during the pandemic, and their association with loneliness varies across age groups and genders. Policy makers and designers of prevention programs must consider age and gender as significant factors that will impact the outcomes of such programs. Parents and teachers with young children can organize more nondigital leisure activities to reduce the potential overuse of digital gadgets used for gaming. Findings from this study may also help parents and teachers to plan family and school activities for children and adolescents. If choices for alternative leisure activities are limited due to public health concerns during the pandemic, parental supervision is very important. Parents need to establish ground rules that limit the time for accessing digital gadgets, help younger ones to set priorities for daily routines, and supervise children so they will not play games during their online classes. If parents are also interested in gaming, they can play together with their children to strengthen parent-child interactions, family relationships, and understanding of their children’s interests and habits.

Since gaming can enhance one’s sense of autonomy, competence, and connectedness with others [12], it should not just be considered as a harmful and addictive behavior. If conducted for the right amount of time and in the appropriate contexts, gaming can be a healthy behavior. It is exciting to learn that some *functional* games have been developed specifically to promote mental well-being during the pandemic [61]. For instance, a game named Shadow’s Edge was developed based on empirical and theory-based foundations, such as narrative therapy and artistic expression, to help players cope with isolation and fear during school closures [62]. Interestingly, it is great to learn that major game publishers around the world and the World Health Organization are supporting the #PlayApartTogether campaign to encourage staying home and maintaining social distance by gaming at home during the lockdown in many countries [63]. In conclusion, gaming can be helpful to the well-being of young people, but can also be harmful, especially to those with lower self-control tendencies. Mature caregivers can help promote positive gaming by providing parental supervision. In addition, more game developers can take steps to design more functional games with social scientists and young people that promote mental well-being and protect players’ health by including planned designs to minimize addictive gaming behaviors.

## Appendix

Multimedia Appendix 1 Supplementary files.

## Abbreviations

aOR: adjusted odds ratio
DSM-5: Diagnostic and Statistical Manual of Mental Disorders, Fifth Edition
GAS: Game Addiction Scale
MSPSS: Multidimensional Scale of Perceived Social Support
OR: odds ratio
SES: socioeconomic status
