# Supplementary material for: Leisure and Problem Gaming Behaviors Among Children and Adolescents During School Closures Caused by COVID-19 in Hong Kong: Quantitative Cross-sectional Survey Study
Source: JMIR Serious Games. 2021 May 7;9(2):e26808. doi: 10.2196/26808 (PMC8108935; doi:10.2196/26808)
Supplement: Multimedia Appendix 1 [file games_v9i2e26808_app1.docx]

**Table S1.** Participant characteristics in different gender and school groups (N = 2863)

| Variable | | Gender | | *P* value | School Groups | | *P* value |
| --- | --- | --- | --- | --- | --- | --- | --- |
|  |  | Male | Female |  | Primary | Secondary |  |
| **Gender ^a^, n (%)** | |  | |  |  |  |  |
|  | Male (n=1346) | N/A | N/A |  | 323 (24.0) | 1023 (76.0) | <.001 |
|  | Female (n=1502) | N/A | N/A |  | 274 (18.2) | 1228 (81.8) |  |
|  | Total (n=2848) | N/A | N/A |  | 597 (21.0) | 2251 (79.0) |  |
| **School Group ^a^, n (%)** | |  | |  |  |  |  |
|  | Primary (n=597) | 323 (54.1) | 274 (45.9) | <.001 | N/A | N/A |  |
|  | Secondary (n=2251) | 1023 (45.4) | 1228 (54.6) |  | N/A | N/A |  |
|  | Total (n=2848) | 1346 (47.3) | 1502 (52.7) |  | N/A | N/A |  |
| **Live with parent ^a^, n (%)** | |  | |  |  |  |  |
|  | Both parents (n=2282) | 1058 (46.4) | 1222 (53.6) | .183 | 488 (21.4) | 1794 (78.6) | .069 |
|  | With father (n=109) | 59 (54.1) | 50 (45.9) |  | 30 (27.5) | 79 (72.5) |  |
|  | With mother (n=324) | 166 (51.2) | 158 (48.8) |  | 59 (18.2) | 265 (81.8) |  |
|  | Neither parent (n=103) | 50 (48.5) | 53 (51.5) |  | 15 (14.6) | 88 (85.4) |  |
|  | Total (n=2818) | 1333 (47.3) | 1483 (52.7) |  | 592 (21.0) | 2226 (79.0) |  |
| **Father’s education level ^a^, n (%)** | |  | |  |  |  |  |
|  | Primary school (n=112) | 63 (56.3) | 49 (43.8) | .011 | 33 (29.5) | 79 (70.5) | <.001 |
|  | Middle school (n=1034) | 471 (45.6) | 563 (54.4) |  | 241 (23.3) | 793 (76.7) |  |
|  | University or above (n=577) | 238 (41.3) | 338 (58.7) |  | 90 (15.6) | 487 (84.4) |  |
|  | Total (n=1723) | 772 (44.8) | 950 (55.2) |  | 364 (21.1) | 1359 (78.9) |  |
| **Mother’s education level ^a^, n (%)** | |  | |  |  |  |  |
|  | Primary school (n=122) | 62 (50.8) | 60 (49.2) | .266 | 32 (26.2) | 90 (73.8) | .023 |
|  | Middle school (n=1075) | 479 (44.6) | 596 (55.4) |  | 233 (21.7) | 842 (78.3) |  |
|  | University or above (n=569) | 243 (42.8) | 325 (57.2) |  | 97 (17.0) | 472 (83.0) |  |
|  | Total (n=1766) | 784 (44.4) | 981 (55.6) |  | 362 (20.5) | 1404 (79.5) |  |
| **Father has a paid job ^a^, n (%)** | |  | |  |  |  |  |
|  | No (n=132) | 66 (50.0) | 66 (50.0) | .241 | 29 (22.0) | 103 (78.0) | .862 |
|  | Yes (n=1905) | 852 (44.7) | 1052 (55.3) |  | 431 (22.6) | 1474 (77.4) |  |
|  | Total (n=2037) | 918 (45.1) | 1118 (54.9) |  | 460 (22.6) | 1577 (77.4) |  |
| **Mother has a paid job ^a^, n (%)** | |  | |  |  |  |  |
|  | No (n=659) | 296 (44.9) | 363 (55.1) | .867 | 132 (20.0) | 527 (80.0) | .210 |
|  | Yes (n=1429) | 647 (45.3) | 781 (54.7) |  | 321 (22.5) | 1108 (77.5) |  |
|  | Total (n=2088) | 943 (45.2) | 1144 (54.8) |  | 453 (21.7) | 1635 (78.3) |  |
| **Family owns car ^a^, n (%)** | |  | |  |  |  |  |
|  | No (n=1848) | 851 (46.1) | 996 (53.9) | .265 | 475 (25.7) | 1373 (74.3) | <.001 |
|  | Yes (n=693) | 336 (48.6) | 356 (51.4) |  | 103 (14.9) | 590 (85.1) |  |
|  | Total (n=2541) | 1187 (46.8) | 1352 (53.2) |  | 578 (22.7) | 1963 (77.3) |  |
| Household appliance ^b^, mean (SD) | | 4.70 (1.58) | 4.88 (1.38) | .001 | 4.58 (1.40) | 4.86 (1.50) | <.001 |
| Learning e-device ^b^, mean (SD) | | 2.51 (1.04) | 2.62 (0.96) | .003 | 2.36 (1.00) | 2.63 (0.99) | <.001 |
| Home Internet access ^b^, mean (SD) | | 2.96 (1.00) | 3.11 (0.86) | <.001 | 3.07 (0.95) | 3.03 (0.93) | .359 |
| Age ^b^, mean (SD) | | 12.58 (1.39) | 12.64 (1.28) | .204 | 10.78 (1.05) | 13.09 (0.92) | <.001 |
| Parental support (MSPSS^c^ family subscale score) ^b^, mean (SD) | | 3.88 (1.30) | 3.93 (1.20) | .256 | 4.13 (1.34) | 3.84 (1.21) | <.001 |
| Parental supervision score ^b^, mean (SD) | | 4.07 (1.32) | 4.30 (1.15) | <.001 | 4.33 (1.33) | 4.15 (1.22) | .003 |
| Loneliness score ^b^, mean (SD) | | 0.44 (0.82) | 0.59 (0.92) | <.001 | 0.51 (0.90) | 0.52 (0.87) | .860 |
| Depression (PHQ-9^d^ score) ^b^, mean (SD) | | 4.65 (4.54) | 6.13 (5.59) | <.001 | 5.09 (5.08) | 5.53 (5.20) | .069 |
| Anxiety (GAD-7^e^ score) ^b^, mean (SD) | | 3.24 (4.33) | 4.53 (5.37) | <.001 | 3.62 (4.77) | 3.98 (4.98) | .115 |

^a^Chi-square was performed to compare differences in gender and age groups with respect to different characteristics.

^b^Independent-Samples T-Test was performed to compare differences in gender and age groups with respect to different characteristics.

^c^MSPSS: Multidimensional Scale of Perceived Social Support.

^d^PHQ-9: Patient Health Questionnaire-9.

^e^GAD-7: Generalized Anxiety Disorder-7.

**Table S2.** Multinomial logistic regression of excessive and pathological game addiction in primary school students (N = 599)

|  | Odds ratio (95% CI) ^a^ | | | |
| --- | --- | --- | --- | --- |
|  | Crude Model ^b^ | Model 1 ^c^ | Model 2 ^d^ | Model 3 ^e^ |
| **Excessive Game Addiction** | | | | |
| Loneliness | 0.98 (0.92-1.05) | 1.01 (0.94-1.08) | 0.94 (0.87-1.01) | 0.71^***^ (0.65-0.78) |
| Age | N/A ^f^ | 0.94^**^ (0.89-0.98) | 0.92^**^ (0.87-0.97) | 0.94^*^ (0.89-0.99) |
| Gender |  |  |  |  |
| Male | N/A | 1.69^***^ (1.50-1.91) | 1.60^***^ (1.42-1.82) | 1.80^***^ (1.58-2.06) |
| Female | N/A | Reference | Reference | Reference |
| Live with parent |  |  |  |  |
| None | N/A | N/A | 0.82 (0.54-1.24) | 0.83 (0.54-1.29) |
| Either father or mother | N/A | N/A | 1.50^***^ (1.27-1.77) | 1.47^***^ (1.24-1.75) |
| Both father and mother | N/A | N/A | Reference | Reference |
| Father has a paid job |  |  |  |  |
| None | N/A | N/A | 1.32^*^ (1.04-1.67) | 1.16 (0.90-1.49) |
| Yes | N/A | N/A | Reference | Reference |
| Mother has a paid job |  |  |  |  |
| None | N/A | N/A | 0.77^***^ (0.67-0.88) | 0.84^*^ (0.72-0.97) |
| Yes | N/A | N/A | Reference | Reference |
| Household appliance | N/A | N/A | 1.04 (0.99-1.09) | 1.03 (0.98-1.08) |
| Learning electronic device | N/A | N/A | 0.76^***^ (0.71-0.82) | 0.77^***^ (0.72-0.83) |
| Home Internet access | N/A | N/A | 0.81^***^ (0.76-0.87) | 0.79^***^ (0.73-0.85) |
| Parental support | N/A | N/A | 0.87^***^ (0.82-0.92) | 0.93^*^ (0.88-0.99) |
| Parental supervision | N/A | N/A | 1.01 (0.96-1.07) | 1.00 (0.94-1.06) |
| Depression | N/A | N/A | N/A | 1.06^***^ (1.04-1.08) |
| Anxiety | N/A | N/A | N/A | 1.05^***^ (1.03-1.07) |
| **Pathological Game Addiction** | | | | |
| Loneliness | 1.49^***^ (1.33-1.67) | 1.58^***^ (1.40-1.78) | 1.45^***^ (1.27-1.66) | 0.94 (0.80-1.12) |
| Age | N/A | 1.06 (0.95-1.17) | 1.05 (0.95-1.17) | 1.01 (0.90-1.13) |
| Gender |  |  |  |  |
| Male | N/A | 2.47^***^ (1.86-3.26) | 2.05^***^ (1.53,2.76) | 2.56^***^ (1.85-3.53) |
| Female | N/A | Reference | Reference | Reference |
| Live with parent |  |  |  |  |
| None | N/A | N/A | 0.83 (0.39-1.77) | 0.94 (0.42-2.07) |
| Either father or mother | N/A | N/A | 1.71^**^ (1.22-1.40) | 1.45 (1.00-2.11) |
| Both father and mother | N/A | N/A | Reference | Reference |
| Father has a paid job |  |  |  |  |
| None | N/A | N/A | 1.61^*^ (1.02-2.53) | 1.59^*^ (1.00-2.52) |
| Yes | N/A | N/A | Reference | Reference |
| Mother has a paid job |  |  |  |  |
| None | N/A | N/A | 0.84 (0.62,1.15) | 0.98 (0.71-1.35) |
| Yes | N/A | N/A | Reference | Reference |
| Household appliance | N/A | N/A | 0.75^***^ (0.68-0.82) | 0.75^***^ (0.68-0.83) |
| Learning electronic device | N/A | N/A | 0.71^***^ (0.60-0.83) | 0.68^***^ (0.58-0.81) |
| Home Internet access | N/A | N/A | 0.62^***^ (0.55-0.71) | 0.61^***^ (0.53-0.70) |
| Parental support | N/A | N/A | 0.82^**^ (0.73-0.93) | 0.93 (0.81-1.06) |
| Parental supervision | N/A | N/A | 1.05 (0.94-1.18) | 0.99 (0.88-1.12) |
| Depression | N/A | N/A | N/A | 1.07^**^ (1.03-1.12) |
| Anxiety | N/A | N/A | N/A | 1.09^***^ (1.05-1.14) |

^a^The odds ratios and 95% CIs for excessive and pathological gaming addiction behaviors were calculated separately with leisure gaming as the reference sample.

^b^In the crude model, we examined the association between loneliness (continual) and gaming.

^c^In Model 1, we additionally adjusted for age and gender.

^d^In Model 2, we additionally adjusted for family structure, the father’s job status-the mother’s job status, household appliances (continual), e-learning devices (continual), home internet access (continual), parental support (continual), and parental supervision (continual).

^e^In Model 3, we additionally adjusted for depression (continual) and anxiety (continual).

^f^N/A: not applicable; this variable was adjusted for in subsequent models.

^*^ *P* < .05; ^**^ *P* < .01; ^***^ *P* < .001.

**Table S3.** Multinomial logistic regression of excessive and pathological game addiction in secondary school students (N = 2264)

|  | Odds ratio (95% CI) ^a^ | | | |
| --- | --- | --- | --- | --- |
|  | Crude Model ^b^ | Model 1 ^c^ | Model 2 ^d^ | Model 3 ^e^ |
| **Excessive Game Addiction** | | | | |
| Loneliness | 1.26^***^ (1.21-1.30) | 1.30^***^ (1.25-1.35) | 1.23^***^ (1.18-1.27) | 1.00 (0.95-1.05) |
| Age | N/A ^f^ | 0.88^***^ (0.85-0.90) | 0.86^***^ (0.83-0.89) | 0.84^***^ (0.81-0.87) |
| Gender |  |  |  |  |
| Male | N/A | 1.42^***^ (1.33-1.52) | 1.38^***^ (1.29-1.48) | 1.60^***^ (1.48-1.73) |
| Female | N/A | Reference | Reference | Reference |
| Live with parent |  |  |  |  |
| None | N/A | N/A | 0.72^***^ (0.60-0.87) | 0.74^**^ (0.61-0.90) |
| Either father or mother | N/A | N/A | 1.21^***^ (1.10-1.32) | 1.18^**^ (1.06-1.30) |
| Both father and mother | N/A | N/A | Reference | Reference |
| Father has a paid job |  |  |  |  |
| None | N/A | N/A | 1.21^**^ (1.07-1.37) | 1.25^**^ (1.09-1.43) |
| Yes | N/A | N/A | Reference | Reference |
| Mother has a paid job |  |  |  |  |
| None | N/A | N/A | 1.14^***^ (1.06-1.22) | 1.17^***^ (1.08-1.26) |
| Yes | N/A | N/A | Reference | Reference |
| Household appliance | N/A | N/A | 1.00 (0.98-1.02) | 0.99 (0.97-1.02) |
| Learning electronic device | N/A | N/A | 0.85^***^ (0.82-0.88) | 0.88^***^ (0.84-0.91) |
| Home Internet access | N/A | N/A | 0.90^***^ (0.86-0.93) | 0.89^***^ (0.86-0.93) |
| Parental support | N/A | N/A | 0.99 (0.96-1.03) | 1.04^*^ (1.00-1.08) |
| Parental supervision | N/A | N/A | 0.98 (0.95-1.02) | 0.98 (0.95-1.01) |
| Depression | N/A | N/A | N/A | 1.11^***^ (1.09-1.12) |
| Anxiety | N/A | N/A | N/A | 0.98^***^ (0.96-0.99) |
| **Pathological Game Addiction** | | | | |
| Loneliness | 1.38^***^ (1.30-1.46) | 1.44^***^ (1.36-1.52) | 1.35^***^ (1.27-1.44) | 0.88^**^ (0.81-0.97) |
| Age | N/A | 1.00 (0.94-1.05) | 0.98 (0.93-1.04) | 0.96 (0.91-1.02) |
| Gender |  |  |  |  |
| Male | N/A | 2.05^***^ (1.83-2.30) | 1.87^***^ (1.66-2.10) | 2.31^***^ (2.01-2.66) |
| Female | N/A | Reference | Reference | Reference |
| Live with parent |  |  |  |  |
| None | N/A | N/A | 0.51^***^ (0.35-0.74) | 0.60^**^ (0.41-0.87) |
| Either father or mother | N/A | N/A | 1.72^***^ (1.49-1.97) | 2.02^***^ (1.74-2.36) |
| Both father and mother | N/A | N/A | Reference | Reference |
| Father has a paid job |  |  |  |  |
| None | N/A | N/A | 1.21 (0.98-1.48) | 1.33^*^ (1.06-1.66) |
| Yes | N/A | N/A | Reference | Reference |
| Mother has a paid job |  |  |  |  |
| None | N/A | N/A | 0.98 (0.87-1.11) | 0.99 (0.86-1.14) |
| Yes | N/A | N/A | Reference | Reference |
| Household appliance | N/A | N/A | 1.04 (1.00-1.08) | 1.02 (0.97-1.07) |
| Learning electronic device | N/A | N/A | 0.75^***^ (0.70-0.80) | 0.78^***^ (0.73-0.84) |
| Home Internet access | N/A | N/A | 0.86^***^ (0.81-0.91) | 0.87^***^ (0.81-0.94) |
| Parental support | N/A | N/A | 1.02 (0.97-1.08) | 1.11^**^ (1.05-1.18) |
| Parental supervision | N/A | N/A | 0.91^**^ (0.86-0.96) | 0.96 (0.91-1.02) |
| Depression | N/A | N/A | N/A | 1.16^***^ (1.14-1.19) |
| Anxiety | N/A | N/A | N/A | 0.99 (0.97-1.01) |

^a^The odds ratios and 95% CIs for excessive and pathological gaming addiction behaviors were calculated separately with leisure gaming as the reference sample.

^b^In the crude model, we examined the association between loneliness (continual) and gaming.

^c^In Model 1, we additionally adjusted for age and gender.

^d^In Model 2, we additionally adjusted for family structure, the father’s job status, the mother’s job status, household appliances (continual), e-learning devices (continual), home internet access (continual), parental support (continual), and parental supervision (continual).

^e^In Model 3, we additionally adjusted for depression (continual) and anxiety (continual).

^f^N/A: not applicable; this variable was adjusted for in subsequent models.

^*^ *P* < .05; ^**^ *P* < .01; ^***^ *P* < .001.

**Table S4.** Multinomial logistic regression of excessive and pathological game addiction in male students (N = 1346)

|  |  | Odds ratio (95% CI) ^a^ | | |
| --- | --- | --- | --- | --- |
|  |  | Crude Model ^b^ | Model 1 ^c^ | Model 2 ^d^ |
| **Excessive Game Addiction** |  |  |  |  |
| Loneliness |  | 1.23^***^ (1.17-1.29) | 1.20^***^ (1.15-1.27) | 0.90^**^ (0.85-0.96) |
| Age |  | N/A ^e^ | 0.90^***^ (0.87-0.92) | 0.90^***^ (0.87-0.92) |
| Live with parent |  |  |  |  |
| None |  | N/A | 0.76^*^ (0.60-0.95) | 0.89 (0.70-1.13) |
| Either father or mother |  | N/A | 1.23^***^ (1.10-1.37) | 1.32^***^ (1.18-1.48) |
| Both father and mother |  | N/A | Reference | Reference |
| Father has a paid job |  |  |  |  |
| None |  | N/A | 1.15 (0.99-1.34) | 1.10 (0.93-1.30) |
| Yes |  | N/A | Reference | Reference |
| Mother has a paid job |  |  |  |  |
| None |  | N/A | 0.94 (0.86-1.03) | 0.96 (0.87-1.05) |
| Yes |  | N/A | Reference | Reference |
| Household appliance |  | N/A | 1.00 (0.98-1.03) | 0.99 (0.96-1.02) |
| Learning electronic device |  | N/A | 0.83^***^ (0.80-0.87) | 0.83^***^ (0.79-0.87) |
| Home Internet access |  | N/A | 0.87^***^ (0.83-0.90) | 0.86^***^ (0.82-0.90) |
| Parental support |  | N/A | 0.99 (0.96-1.03) | 1.04 (1.00-1.08) |
| Parental supervision |  | N/A | 0.95^**^ (0.91-0.98) | 0.94^**^ (0.90-0.97) |
| Depression |  | N/A | N/A | 1.10^***^ (1.09-1.12) |
| Anxiety |  | N/A | N/A | 1.02^**^ (1.01-1.04) |
| **Pathological Game Addiction** | | | | |
| Loneliness |  | 1.34^***^ (1.25-1.44) | 1.26^***^ (1.17-1.36) | 0.90^*^ (0.81-1.00) |
| Age |  | N/A | 1.09^***^ (1.04-1.14) | 1.09^**^ (1.04-1.15) |
| Live with parent |  |  |  |  |
| None |  | N/A | 1.04 (0.74-1.46) | 1.37 (0.96-1.94) |
| Either father or mother |  | N/A | 1.67^***^ (1.42-1.97) | 1.75^***^ (1.46-2.10) |
| Both father and mother |  | N/A | Reference | Reference |
| Father has a paid job |  |  |  |  |
| None |  | N/A | 1.01 (0.79-1.29) | 0.95 (0.72-1.25) |
| Yes |  | N/A | Reference | Reference |
| Mother has a paid job |  |  |  |  |
| None |  | N/A | 0.96 (0.83-1.12) | 1.02 (0.86-1.20) |
| Yes |  | N/A | Reference | Reference |
| Household appliance |  | N/A | 0.96 (0.92-1.01) | 0.94^**^ (0.89-0.98) |
| Learning electronic device |  | N/A | 0.75^***^ (0.69-0.80) | 0.71^***^ (0.65-0.77) |
| Home Internet access |  | N/A | 0.84^***^ (0.78-0.90) | 0.85^***^ (0.79-0.92) |
| Parental support |  | N/A | 0.98 (0.92-1.04) | 1.03 (0.96-1.11) |
| Parental supervision |  | N/A | 1.01 (0.95-1.07) | 1.05 (0.98-1.12) |
| Depression |  | N/A | N/A | 1.14^***^ (1.12-1.17) |
| Anxiety |  | N/A | N/A | 1.01 (0.99-1.04) |

^a^The odds ratios and 95% CIs for excessive and pathological gaming addiction behaviors were calculated separately with leisure gaming as the reference sample.

^b^In the crude model, we examined the association between loneliness (continual) and gaming.

^c^In Model 1, we additionally adjusted for age, family structure, the father’s job status. the mother’s job status, household appliances (continual), e-learning devices (continual), home internet access (continual), parental support (continual), and parental supervision (continual).

^d^In Model 2, we additionally adjusted for depression (continual) and anxiety (continual).

^e^N/A: not applicable; this variable was adjusted for in subsequent models.

^*^ *P* < .05; ^**^ *P* < .01; ^***^ *P* < .001.

**Table S5.** Multinomial logistic regression of excessive and pathological game addiction in female students (N = 1502)

|  |  | Odds ratio (95% CI) ^a^ | | |
| --- | --- | --- | --- | --- |
|  |  | Crude Model ^b^ | Model 1 ^c^ | Model 2 ^d^ |
| **Excessive Game Addiction** |  |  |  |  |
| Loneliness |  | 1.21^***^ (1.16-1.26) | 1.10^***^ (1.05-1.15) | 0.93^*^ (0.87-0.99) |
| Age |  | N/A ^e^ | 0.91^***^ (0.88-0.94) | 0.88^***^ (0.85-0.91) |
| Live with parent |  |  |  |  |
| None |  | N/A | 0.66^**^ (0.52-0.86) | 0.59^***^ (0.45,0.78) |
| Either father or mother |  | N/A | 1.31^***^ (1.17-1.48) | 1.13 (0.98-1.29) |
| Both father and mother |  | N/A | Reference | Reference |
| Father has a paid job |  |  |  |  |
| None |  | N/A | 1.29^**^ (1.10-1.52) | 1.34^**^ (1.12-1.60) |
| Yes |  | N/A | Reference | Reference |
| Mother has a paid job |  |  |  |  |
| None |  | N/A | 1.21^***^ (1.10-1.32) | 1.29^***^ (1.17-1.42) |
| Yes |  | N/A | Reference | Reference |
| Household appliance |  | N/A | 1.04^*^ (1.00-1.07) | 1.04^*^ (1.00-1.07) |
| Learning electronic device |  | N/A | 0.85^***^ (0.81-0.89) | 0.89^***^ (0.85-0.94) |
| Home Internet access |  | N/A | 0.89^***^ (0.85-0.94) | 0.88^***^ (0.84-0.93) |
| Parental support |  | N/A | 0.90^***^ (0.86-0.94) | 0.96 (0.91-1.01) |
| Parental supervision |  | N/A | 1.07^**^ (1.02-1.12) | 1.06^*^ (1.01-1.12) |
| Depression |  | N/A | N/A | 1.09^***^ (1.08-1.11) |
| Anxiety |  | N/A | N/A | 0.97^**^ (0.96-0.99) |
| **Pathological Game Addiction** | | | | |
| Loneliness |  | 1.61^***^ (1.49-1.73) | 1.42^***^ (1.31-1.54) | 0.83^**^ (0.74-0.93) |
| Age |  | N/A | 1.04 (0.98-1.11) | 0.94 (0.88-1.01) |
| Live with parent |  |  |  |  |
| None |  | N/A | 0.03^**^ (0.01-0.24) | 0 |
| Either father or mother |  | N/A | 1.71^***^ (1.39-2.10) | 2.09^***^ (1.67-2.62) |
| Both father and mother |  | N/A | Reference | Reference |
| Father has a paid job |  |  |  |  |
| None |  | N/A | 1.51^**^ (1.14-2.01) | 2.05^***^ (1.52-2.76) |
| Yes |  | N/A | Reference | Reference |
| Mother has a paid job |  |  |  |  |
| None |  | N/A | 1.00 (0.83-1.20) | 1.01 (0.82-1.25) |
| Yes |  | N/A | Reference | Reference |
| Household appliance |  | N/A | 1.06 (1.00-1.14) | 1.04 (0.97-1.12) |
| Learning electronic device |  | N/A | 0.77^***^ (0.70-0.86) | 0.89^*^ (0.80-0.99) |
| Home Internet access |  | N/A | 0.72^***^ (0.66-0.79) | 0.69^***^ (0.63-0.77) |
| Parental support |  | N/A | 0.96 (0.88-1.05) | 1.08 (0.98-1.19) |
| Parental supervision |  | N/A | 0.81^***^ (0.75-0.89) | 0.83^***^ (0.75-0.91) |
| Depression |  | N/A | N/A | 1.13^***^ (1.10-1.16) |
| Anxiety |  | N/A | N/A | 1.04^*^ (1.01-1.07) |

^a^The odds ratios and 95% CIs for excessive and pathological gaming addiction behaviors were calculated separately with leisure gaming as the reference sample.

^b^In the crude model, we examined the association between loneliness (continual) and gaming.

^c^In Model 1, we additionally adjusted for age, family structure, the father’s job status, the mother’s job status, household appliances (continual), e-learning devices (continual), home internet access (continual), parental support (continual), and parental supervision (continual).

^d^In Model 2, we additionally adjusted for depression (continual) and anxiety (continual).

^e^N/A: not applicable; this variable was adjusted for in subsequent models.

^*^ *P* < .05; ^**^ *P* < .01; ^***^ *P* < .001.
